# Data-driven computational intelligence applied to dengue outbreak forecasting: a case study at the scale of the city of Natal, RN-Brazil

**DOI:** 10.1038/s41598-022-10512-5

**Published:** 2022-04-21

**Authors:** Ignacio Sanchez-Gendriz, Gustavo Fontoura de Souza, Ion G. M. de Andrade, Adrião Duarte Doria Neto, Alessandre de Medeiros Tavares, Daniele M. S. Barros, Antonio Higor Freire de Morais, Leonardo J. Galvão-Lima, Ricardo Alexsandro de Medeiros Valentim

**Affiliations:** 1 Laboratory for Technological Innovation in Health (LAIS), Hospital Universitário Onofre Lopes, Federal University of Rio Grande Do Norte (UFRN), Natal, Rio Grande do Norte Brazil; 2 Advanced Nucleus of Technological Innovation (NAVI), Federal Institute of Rio Grande Do Norte (IFRN), Natal, Rio Grande do Norte Brazil; 3 Department of Computer Engineering and Automation, UFRN, Natal, Rio Grande do Norte Brazil; 4 Municipal Health Department, Zoonoses Control Center, Natal, Rio Grande do Norte Brazil

**Keywords:** Data mining, Data processing

## Abstract

Dengue is recognized as a health problem that causes significant socioeconomic impacts throughout the world, affecting millions of people each year. A commonly used method for monitoring the dengue vector is to count the eggs that Aedes aegypti mosquitoes have laid in spatially distributed ovitraps. Given this approach, the present study uses a database collected from 397 ovitraps allocated across the city of Natal, RN—Brazil. The Egg Density Index for each neighborhood was computed weekly, over four complete years (from 2016 to 2019), and simultaneously analyzed with the dengue case incidence. Our results illustrate that the incidence of dengue is related to the socioeconomic level of the neighborhoods in the city of Natal. A deep learning algorithm was used to predict future dengue case incidence, either based on the previous weeks of dengue incidence or the number of eggs present in the ovitraps. The analysis reveals that ovitrap data allows earlier prediction (four to six weeks) compared to dengue incidence itself (one week). Therefore, the results validate that the quantification of *Aedes aegypti* eggs can be valuable for the early planning of public health interventions.

## Introduction

Dengue is recognized as the most severe human disease caused by an arbovirus. The vector for the disease—as it is for yellow fever, chikungunya, and Zika virus—is the mosquito *Aedes aegypti*^1^. Researches estimate that 10 thousand deaths and 100 million symptomatic infections occur each year in approximately 125 countries^2,3^; whereas Brazil accounted for 55% of cases reported in the Americas in the last three decades^4^. Then, monitoring and controlling Aedes infestation is a valuable public health action to prevent dengue outbreaks.

Moreover, an efficient way for monitoring levels of Aedes is through ovitraps^5,6^, special containers built to collect mosquito eggs^6^. Counting the eggs deposited in spatially distributed ovitraps can serve as a proxy for levels of Aedes infestation, and it allows to determine the vector's geographic distribution, density, and seasonality^7^. Although an ovitrap does not constitute a direct measurement for adult mosquito density, it can work as a good estimator^6,8^.

Notwithstanding, studies using ovitrap data for direct prediction of dengue incidence are yet scarce^8^. Accordingly, a disparity can be observed in studies that address weekly dengue time series forecasting. The reasons for that might be the multifaceted dynamics of the disease itself and the complex associations between mosquito incidence and the risk of infections. Specifically, it has been reported that dengue incidence seems to be influenced by the exposure to areas where contact with infected mosquitos is probable, regardless of the distance of subjects' residence to those places^9^. Although the associations between dengue incidence and socioeconomic status have been addressed elsewhere^10–13^, studies for specific cities may shed light on these complex connections.

The main objectives of the present work are twofold. The first is to extract understandings on dengue disease from complete four-year data, weekly sampled at the city of Natal, located in the State of Rio Grande do Norte (RN), Brazil. The second goal is to train models that allow dengue forecasting for Natal, both by using past samples of dengue cases or previous values of *Aedes aegypti* eggs count (ovitrap data).

In order to address the second goal, we explored the current body of literature to verify methods that have been used for dengue time series prediction. As a result, we found several candidate models, such as Random Forest^14^, Support Vector Regression and LASSO Regression^15^, Autoregressive Integrated Moving Average (ARIMA)^16^ and Seasonal-ARIMA (SARIMA) models^17^. Also, in an extensive literature review^18^, Sylvestre and colleagues analyzed papers published between January 1, 2000 and August 31, 2020. They concluded that the models with the best performances for dengue prediction were Neural Networks and Decision Trees, followed by Support Vector Machines.

In our study, we opted out to use Long Short-Term Memory (LSTM), a neural network model that has been used elsewhere^19–22^ for dengue time series forecasting, outperforming traditional methods such as Random Forest and Lasso Regression^19^. Thus, the novelty of our study related to dengue time series forecasting lies in applying ovitrap data as a predictor in conjunction with the LSTM model.

We have analyzed data of spatially distributed ovitraps and dengue incidence reported by neighborhoods. A compilation of the relevant results obtained in the study is summarized below:

- It was estimated a 1-year seasonality for dengue incidence and vector incidence (quantified through egg deposition) in the data analyzed.
- The estimated time lag between vector and dengue was four weeks.
- In Natal, dengue incidence reveals a strong association with neighborhood socioeconomic status.
- Using dengue cases reported from previous weeks to forecast dengue incidence for the subsequent week allowed us to train LSTM models that show encouraging performance (goodness-of-fit estimated by a correlation coefficient of 0.92).
- Forecasting dengue incidence with ovitrap data as a predictor has indicated a performance (goodness-of-fit estimated by a correlation coefficient of 0.87) similar to that of using dengue incidence.
- The benefit of using ovitrap data is the possibility of earlier detection (six to four weeks in advance) of dengue outbreaks than when the number of dengue cases is used as a predictor (one week in advance).
- Accumulated values for 1-year duration temporal windows have shown a significant association between ovitrap data and dengue incidence.

Such findings underscore the relevance of ovitrap for vector monitoring and planning actions and public health interventions at the municipal level. Additionally, the use of Deep Learning (DL) models and data mining approaches could substantially contribute to epidemiologists and public health specialists to overcome and manage dengue-related problems.

## Results

### Heat map representation of egg density index (EDI) and dengue incidence, grouped by districts

**Figure 1:**
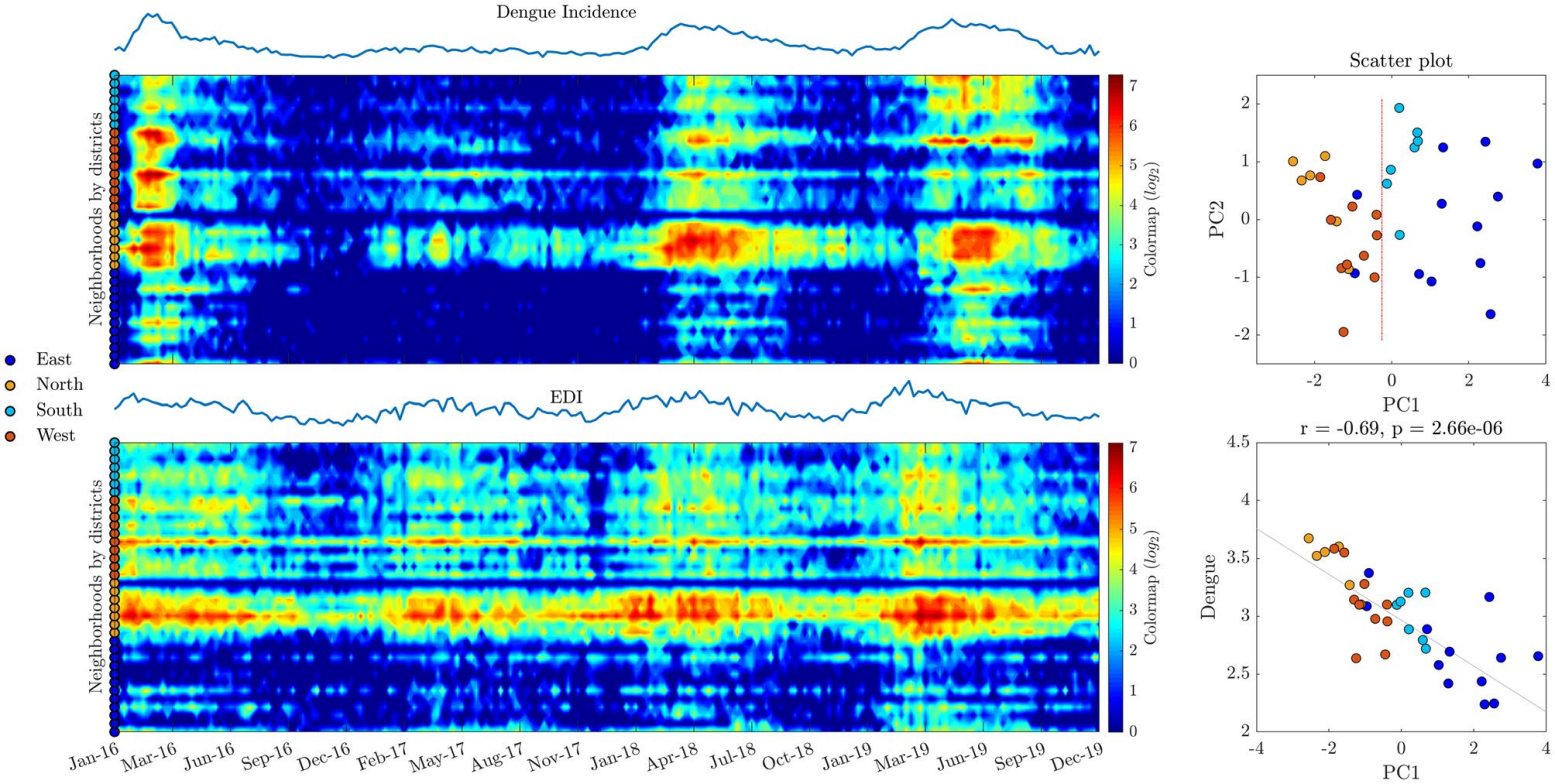
Evolution of dengue incidence and EDI* and the association of accumulated dengue incidence by neighborhoods and socioeconomic variables. (**a**) Representation of heat maps for EDI and dengue incidence, neighborhoods grouped by districts. (**b**) Upper panel: scatter plot of PC1 vs. PC2 for PCA of socioeconomic variables; bottom panel: PC1 vs. accumulated dengue incidence (log2 transformed) by neighborhoods. (*EDI for each neighborhood was weighted by the respective population. The neighborhood with the higher population was multiplied by 1, and the remainder was multiplied by a proportional factor.)

Figure 1 depicts, in the left panels, heat maps for EDI (bottom) and dengue incidence (upper) for neighborhoods grouped by districts. Some observations can be highlighted from the visual inspection of such heat maps. First, an annual seasonal variation seems to be apparent both for dengue incidence and EDI data. Second, trends of EDI increase appear in antecedence of dengue incidence increase. Third, grouping neighborhoods by districts revealed differences among them. For instance, between East and South, as these were the districts with less dengue incidence. In contrast, North and West had higher cases throughout the studied interval.

In the right panels of Fig. 1, two different scatter plots can be observed. The right upper panel represents Principal Component Analysis (PCA) obtained from socioeconomic variables (total income, population, income per capita; see more details in the Methods section). The first and second components (PC1 and PC2) explain 100% of the data variance. The left bottom panel is obtained by a scatter plot between accumulated dengue incidence and the first component (PC1, 61.82% of the explained variance) obtained for the PCA projection cited above. A Pearson's correlation coefficient computed between PC1 and dengue incidence (transformed by log10 operation), shows a significant negative association between the variables (r = −0.69, *p* < 0.001). A plot of the values of income per capita for each neighborhood shows that income is utterly perfectly distributed into two different groups: East-South (Group 1) and North-West (Group 2) (see Supplementary Fig. 1). The exceptions are the low-income neighborhoods "Mãe Luiza" and "Alecrim", which regionally belong to the East-South group but have socioeconomic profiles compatible with the North-West group. Supplementary Fig. 1 also depicts a negative association between dengue incidence and income when analyzed by districts; specifically, districts with the lowest income per capita have the highest incidence.

### Seasonality and lag between dengue and ovitrap data, quantified by discrete fourier transform and cross-correlation

The discrete Fourier transform (DFT) of the mean values for dengue incidence and EDI were calculated to estimate the periodicity of both time series, respectively (see Supplementary Fig. 2). The peak of DFT for both analyzed time series estimated a seasonality of 52 weeks, which coincided with the number of epidemiological weeks for one year. Thus, results indicate a 1-year periodicity for dengue incidence and vector (egg density) at a city level. In addition, cross-correlation was used to estimate the time lag between mean EDI and mean dengue incidence. The time lag estimated by cross-correlation resulted in four weeks. Hence, these results suggest that an increase in EDI precedes dengue incidence increase in approximately one month at the city level.

### Predicting aggregate dengue incidence for the city of Natal

The time series for dengue incidence and EDI for all neighborhoods were aggregated, thus resulting in a two time series, respectively, which could be used as a global indicator for dengue occurrence and Aedes incidence for Natal. In this case, we have trained LSTM models for forecasting aggregate dengue values for the whole municipality. As predictors, it was used either aggregate dengue values or aggregate EDI values. The models were trained with the following samples of the time series used as a predictor (referencing $i$ the target sample of the dengue time series):

- $i-1$ previous sample (1 past sample),
- $i-3,i-2,i-1$ previous samples (3:1 past samples),
- $i-4,i-3,i-2$ previous samples (4:2 past samples),
- $i-5,i-4,i-3$ previous samples (5:3 past samples),
- $i-6,i-5,i-4$ previous samples (6:4 past sample).

These models were named with the nomenclature listed below:

- ${D}_{i-j}\to {D}_{i}$: dengue ($i-j$ past samples) for predicting dengue at the target week $i$.
- ${O}_{i-j}\to {D}_{i}$: ovitrap index ($i-j$ past samples) for predicting dengue at the target week $i$.

**Figure 2:**
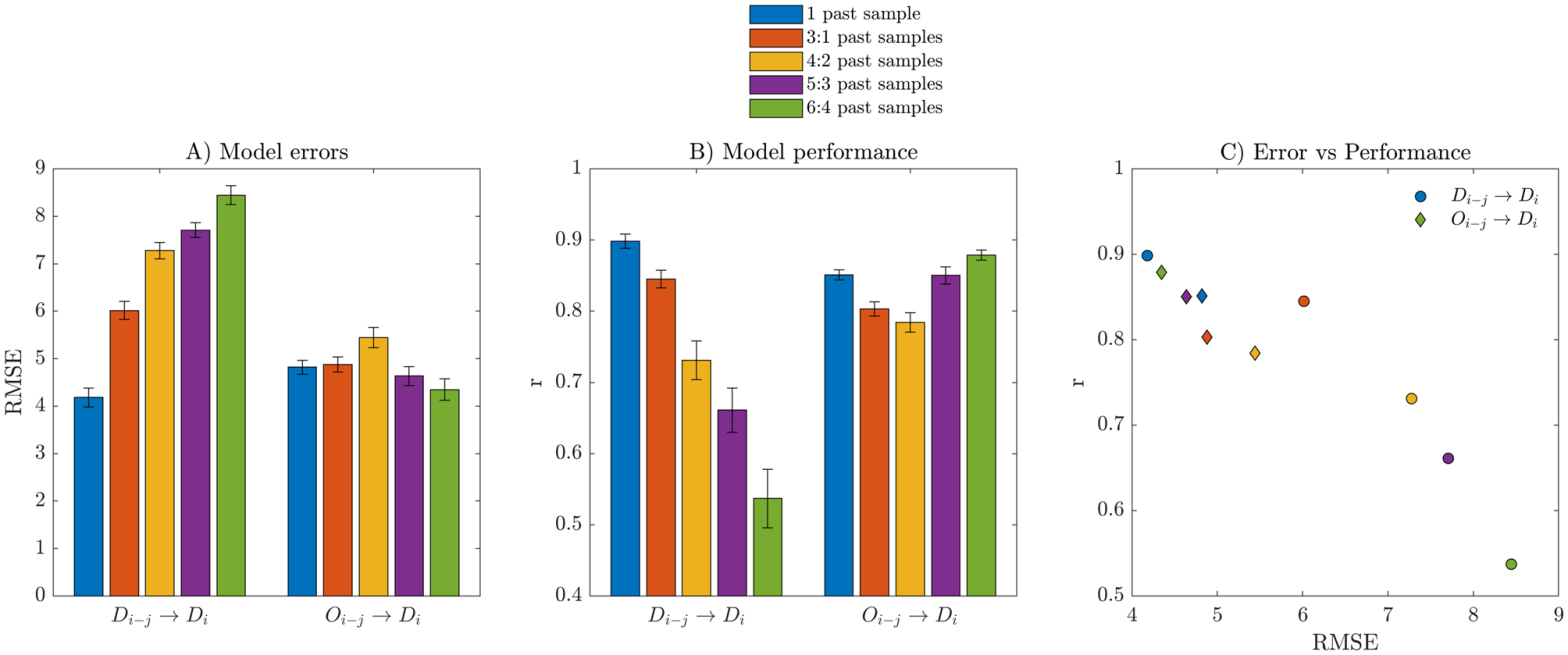
Evaluation of LSTM models performance for dengue forecasting based on aggregate time-series data. The models were trained and tested 30 times. Bars indicate mean values, and whiskers indicate a standard error, both for RMSE and r metrics.

Figure 2 illustrates the performance of the trained models of dengue forecasting for aggregate values.

The error of the models was quantified by RMSE (Root-mean-square Error) and the goodness-of-fit by correlation coefficient (r) between observed values and predicted values. The plot RMSE versus r indicates that the two best-ranked models were D → D, when the input considers the $i-1$ previous sample, and O → D, when the input considers $i-6,i-5,i-4$ previous samples. Also, it can be observed from Fig. 2 that the performance of the models D → D decreases when older samples are used for prediction. In contrast, this was not the case for O → D models, where the best performance was achieved with the older samples of the predictor. Finally, it is worth noting that the RMSE for the O → D models is lower than the D → D models' values, mainly when older samples are used for prediction.

**Figure 3:**
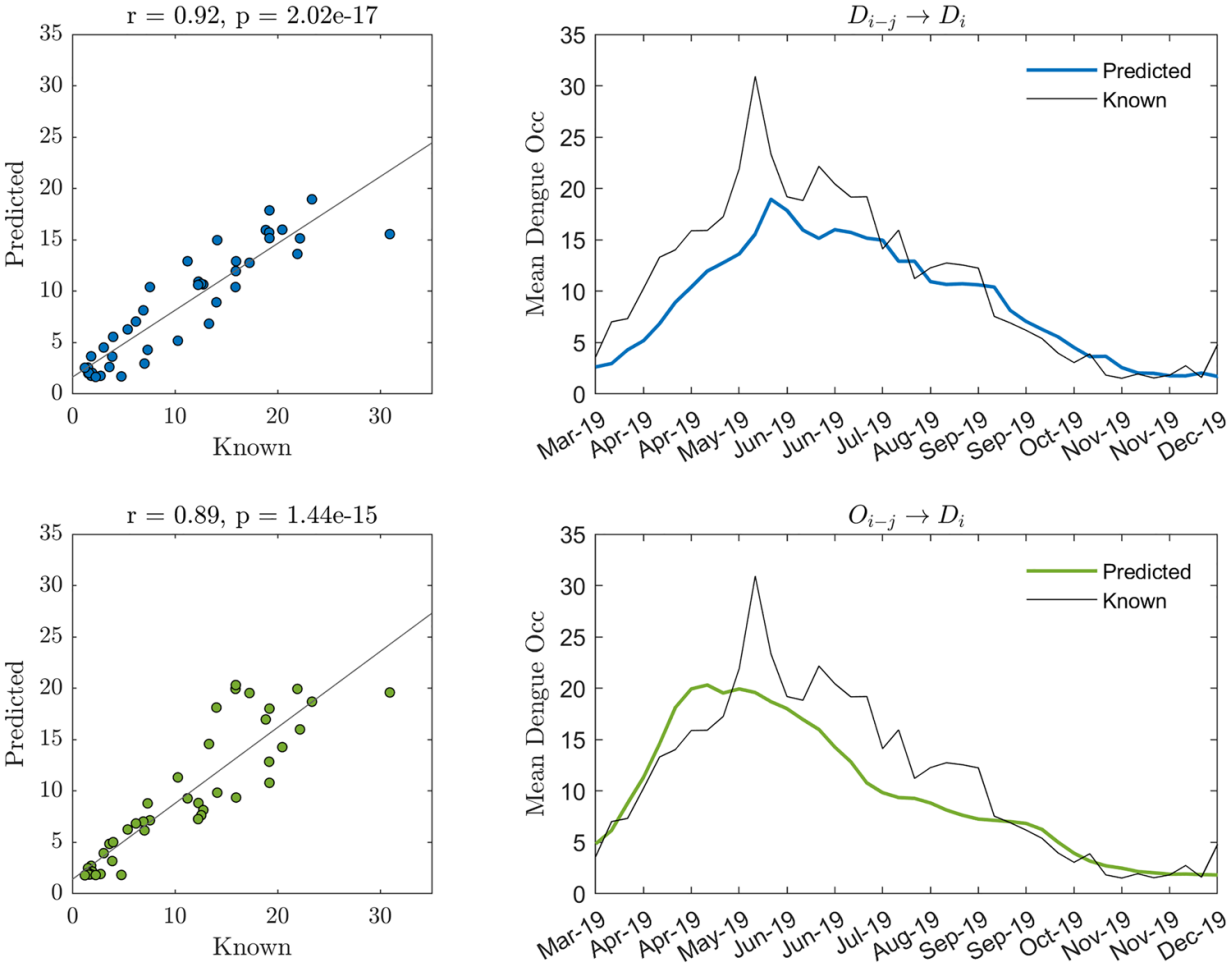
Predictions of LSTM models were trained and tested 30 times. Left panels depict the scatter plots for Observed vs. Predicted values. The right panels show the time series of the mean of the predicted values for the 30 repetitions and the observed values for a test set of samples.

A detailed perspective of the response of the two models with the best predictions is represented in Fig. 3. The predicted response for these models closely follows the actual values, which is quantified by a correlation coefficient close to 0.9 (0.92 and 0.89 respectively, *p* < 0.001) and RMSE < 5 in both cases.

### Analyzing aggregate values broken down by yearly periods

**Figure 4:**
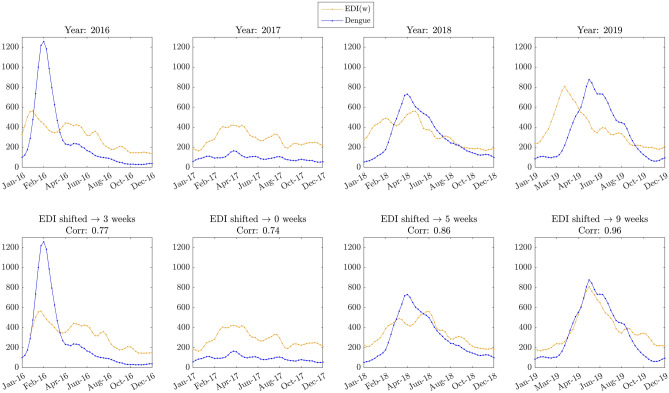
Dengue and EDI by annual periods. Upper panels show Dengue Incidence and EDI aggregated for the municipality; the time series are categorized yearly, from 2016 to 2019. The bottom panels illustrate Dengue Incidence and EDI, but the EDI time series was shifted for visualizing the best correlation. For all panels, aggregated EDI was estimated by weighting the values for the neighborhoods by the respective population. The neighborhood with the higher population was multiplied by 1, and the remainder was multiplied by a proportional factor.

Figure 4 reveals that time lag and cross-correlations could change over the years, suggesting that a complex dynamic could trigger underlying links between the vector increase estimated by ovitraps and the incidence of dengue. Of note, not every year in which an increase in egg density occurred, there was an increase in dengue cases (2017). However, those years in which there was an increase in dengue cases were preceded by increased egg count in ovitraps.

**Figure 5:**
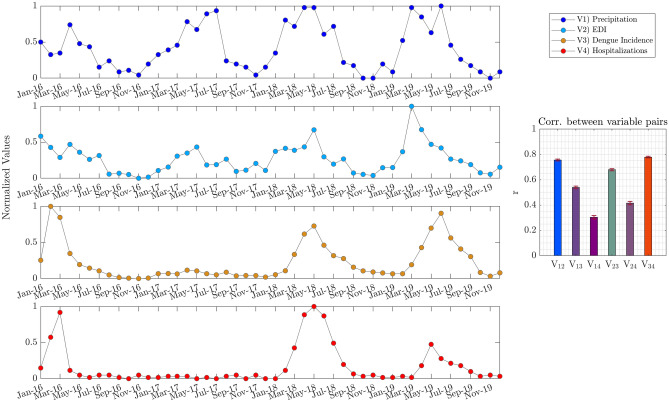
Time series by monthly accumulated values and its pairwise associations. The time series represents the normalized values (0–1 scaling) of Precipitation, EDI, Dengue Incidence, and Hospitalizations, each colored differently. The right panel represents a pairwise similarity among the analyzed variables; the similarity was estimated by computing the correlation coefficient between randomly selected samples (100 times); the bar height represents mean values, and whiskers represent standard errors.

Such results encouraged new analyses, aiming to explore other factors related to dengue incidence in Natal. Thus, other data, as precipitations and dengue hospitalizations, were included in the analysis. Since dengue hospitalizations are registered at a monthly sampling rate, all analyzed time series were clustered by month. Then, the normalized values for Precipitations (V1), EDI (V2), Dengue Incidence (V3), and Dengue Hospitalizations (V4) are depicted in Fig. 5. From Fig. 4, it is worth noting that the time series pairs ${V}_{34}$ (Dengue Incidence and Hospitalizations) and ${V}_{12}$ (Precipitation and EDI) seem to present similar patterns of evolution through the four years. The similarity between the mentioned time series was estimated by computing the correlation coefficient between randomly selected samples (100 times) for all possible pairwise combinations of the four-time series. For more details about the similarity estimation mentioned above, see the Methods section. Finally, the estimation between the time series pairs was plotted in a bar graph, also included in Fig. 5. As expected, the pairs ${V}_{34}$ and ${V}_{12}$ registered the higher score for similarity, followed by the similarity of the pair ${V}_{23}$ (EDI vs. Dengue Incidence).

**Figure 6:**
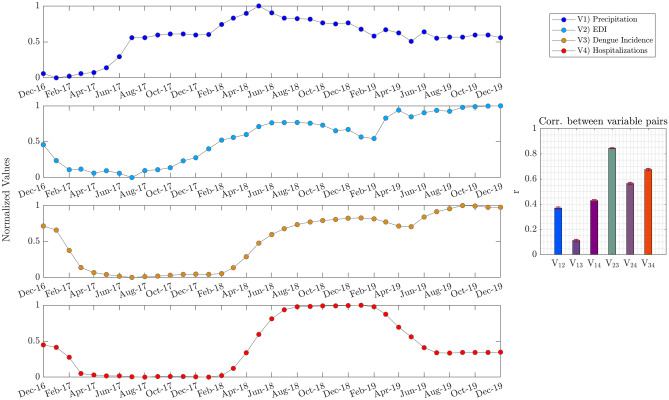
Accumulated values for 1-year sliding windows and their pairwise relations. The time series represents the normalized values (0–1 scaling) of the Precipitation, EDI, Dengue Incidence, and Hospitalizations, each colored differently. The right panel represents a pairwise similarity between the analyzed variables; the similarity was estimated by computing the correlation coefficient between samples selected randomly (100 times); the bar height represents mean values, and whiskers represent standard errors.

However, if we observe the time series plotted in Fig. 5, some interesting points can be highlighted. For instance, one might ask whether the years with higher values for a given time series correspond to similar periods for the other time series. It can be noticed that for V3 and V4, this statement is not fulfilled. The year with the highest accumulates for dengue incidence is 2019, since, for dengue hospitalizations, the highest accumulates were reached in 2018 (see Supplementary Fig. 3). To further expand this analysis, we computed the accumulated values for 1-year sliding temporal windows using a one-month step for all time series represented in Fig. 5. As a result, another four time series were obtained and plotted in Fig. 6. By applying the accumulated values for one-year sliding windows, all pairwise combinations' similarity was also estimated and included as a bar graph (see Fig. 6). The analysis based on the yearly accumulated values presented in Fig. 6 indicates that pair ${V}_{23}$ (EDI vs. dengue incidence) have the highest similarity, followed by pairs ${V}_{34}$ (dengue incidence vs. dengue hospitalizations) and ${V}_{24}$ (EDI vs. dengue hospitalizations). Another considerable aspect of the time series in Fig. 6 is that an increase in precipitation precedes an increase in EDI, which precedes dengue incidence. Hence, it seems to be corresponding to dengue hospitalizations.

## Discussions

### Seasonality and time lag

The 1-year seasonality detected from a visual inspection of heat maps represented in Fig. 1 was verified by calculating the DFT of the mean values of dengue incidence and EDI. This periodicity for the disease to occur and the *Aedes aegypti* population have been reported elsewhere^23–26^. However, yearly periodicities for dengue cases do not necessarily imply that all years have the equivalent incidence level. For instance, in 2017, the levels of dengue incidence for all Natal neighborhoods were considerably lower than the other recorded years. This fact could be related to several aspects, such as the presence of susceptible people for the predominant circulating serotype^27^ since dengue is caused by four different virus serotypes^28^. In addition, other aspects such as the complex interaction between environmental drivers and the four dengue serotypes could occur^29^. Also, the reported incidence of dengue cases in 2017 was lower than expected for Brazil and Colombia^30^. That could be related to the previous human population infected with the Zika virus in those regions.

The cross-correlation estimates one month of the time lag between EDI and dengue incidence, which is consistent with the expected elapsed time from mosquito eggs depositions to the adult phase and subsequent virus transmission to humans. Similar results have been reported in the literature^8^. The high correlation and the possibility of anticipation of the epidemic's severity with a time-lapse of four weeks make ovitrap monitoring extraordinarily important for the timely adoption of contingency measures against dengue. In addition, it contributes to the early detection of the epidemic, thus aiding its controllability.

Concurrently, the significant correlation found at the city level is not necessarily expected at the local neighborhood level^8^. This fact suggests that dengue is a disease of eminently municipal scope. Then, it points to the demand for systemically confronting dengue in the territory where a given community lives, not only at the local level, where houses are located.

### Dengue and socioeconomic status in the city of Natal

The heat maps in Fig. 1 illustrate that neighborhoods from North and West districts face the highest dengue occurrence. By contrast, neighborhoods from the East and South regions have the lowest incidence. Thus, the city of Natal is stratified into neighborhoods with a notably divergent socioeconomic status, which can be observed in Supplementary Fig. 1. In addition, it can be pointed out that poor districts suffer from a higher incidence of dengue. These results coincide with previous studies suggesting that dengue incidence is correlated with lower socioeconomic status^11^. Also, studies report that poverty could be related to factors that increase the risk of human exposure to *Aedes aegypti*^26^.

Besides, the visualization based on heat maps such as those presented in Fig. 1 offers insights on the dynamics and evolution of vector and dengue incidence by localities (in this case, neighborhoods) through a long-term registered period.

### Performance of models trained for predicting aggregate values

Deep learning (DL) has been applied in several areas of research in the last decade, with extraordinary results. Here we applied LSTM models. Models trained using dengue as a predictor for predicting values for the next week (D → D) obtain the best results among all tested models. However, it is relevant to note that using ovitrap data for predicting aggregated Dengue Incidence (O → D) shows similar performance to D → D. This suggests the relevance of egg monitoring at the global scale of the city. The performance obtained for aggregated values remarks the usefulness of ovitraps for planning health actions at the city level. One can note that although D → D shows better performance, O → D shows comparable results, but with the advantage of anticipation. By using EDI time series as a predictor, the best performance was obtained for six to four weeks before the target week for dengue incidence. These facts highlight the importance of ovitrap monitoring for early epidemic risk detection and, therefore, point to the possibility of delineating health actions to prevent dengue outbreaks.

As opposed to the conventional way of being perceived as underlying local and peridomiciliary conditions of infection, the dynamics of the spread of dengue fever has shown to be a result of municipal dynamics, probably produced by urban mobility of people but also of the infected vector itself. This urban mobility is crucial for the set of requirements for the outbreak of a dengue epidemic to initiate^22,31^.

Therefore, the local variables of vector infestation and increased eggs in the ovitraps should be understood, not as local but as municipal triggers. Thus, it can interconnect regions with large numbers of vectors and little viral circulation with those where viral circulation is already established even though there is a low infestation of mosquitoes.

The nexus between the set of conditions for the epidemic outbreak generated locally will be consummated through urban living, characterized by a community whose life and work practices take place without defined territorial limits within a municipality, marked by urban mobility. Therefore, the local infestation interests the city as a whole in a complex dynamic whose practices juxtapose associations within the epidemiological chain, turning the local risk into a municipal risk.

### Accumulated values for 1-year sliding windows

Comparing the time series for accumulated values obtained for 1-year sliding windows provides two compelling results subsequently discussed. First, when analyzing the time series Precipitation (V1), EDI (V2), Dengue Incidence (V3), and Dengue Hospitalizations (V4), the higher correlations were obtained from pair V_12_ and pair V_34_ (see Fig. 5). These connections are consistent with expectations since precipitation creates favorable conditions for *Aedes aegypti* reproduction, and higher dengue incidence favors probabilities of hospitalizations. Nevertheless, a subtle association appears when analyzing the respective time series for the accumulated values for a 1-year window. That is, the most significant correlation emerges between EDI and Dengue Incidence for yearly accumulates. This suggests that higher accumulations of *Aedes aegypti* eggs over 1-year periods are strongly associated with dengue incidence. Once again, it reinforces egg monitoring as a relevant variable. But this time from a long-term perspective.

Second, Fig. 6 also suggests that the increase in yearly accumulated precipitation precedes an increase in accumulated egg depositions (measured by EDI), which precedes dengue incidence and hospitalizations.

## Overall considerations

### Concluding remarks

Our study explored a four-year dataset composed of *Aedes aegypti* eggs counted for 397 spatially distributed ovitraps in the city of Natal, weekly sampled. Moreover, we analyzed the dengue incidence reported for Natal's neighborhoods. Dengue incidence of these neighborhoods shows a positive association with socioeconomic indicators of poverty. Yearly trends were quantified for vector and dengue incidence, and a time lag of four weeks was estimated between these variables. Thus, early detection of dengue outbreaks may be possible through ovitrap data four to six weeks in advance. Accumulated values for annual temporal windows evidence a robust correlation between *Aedes aegypti* egg depositions and dengue incidence in Natal. Our work shows the significance of continuous recording of dengue incidence for long periods and reinforces the relevance of ovitrap monitoring. From a broader point of view, the results presented here complement previous studies focused on the prediction of diseases such as rabies, influenza, and malaria^32–34^. Taken together, these works reinforce the potential of using machine learning and data mining as innovative and powerful tools to predict different diseases. This is particularly important as it allows strategic intervention based on real-time data in a specific territory before disease outbreaks occur.

### Future work

Further studies approaching dengue prediction should include human mobility data as a predictor and circulating serotypes. Our current study mainly focused on the advantages of using ovitrap data to predict dengue incidence and enable earlier public health interventions. Although we have not analyzed human mobility data, the literature review performed during our research identified it as a factor used for dengue forecasting. Based on our results, we believe that the information mined from ovitrap data could considerably contribute to improving dengue prediction tasks with other variables, e.g., human mobility. In addition**,** it would be opportune for upcoming researches to apply methods for exploring causal connections between vector proliferation and dengue incidence. Finally, subsequent studies may focus on vector incidence forecasting and use prediction to support and plan actions for controlling *Aedes aegypti* vector proliferation.

## Methods

### Database description

The incidence of dengue cases registered in each neighborhood of Natal city, weekly sampled (52 epidemiological weeks a year) between 2016 and 2019), was used as the target for forecasting. The source for dengue data was the Notifiable Diseases Information System (SINAN, according to the acronym in Portuguese). Also, ovitrap egg counts for *Aedes aegypti*, collected every week from 397 ovitraps and reported by the Zoonoses Center of Natal, were used in the study. See Supplementary Fig. 5 for the geographical distribution of ovitraps at Natal municipality. The database as well as the code for prediction models are publicly available online (see Data and code availability section).

### Ovitrap indexes

Ovitrap Positivity Index (OPI) and Egg Density Index (EDI) are entomological indices commonly used for *Aedes aegypti* monitoring. The OPI is defined as the ratio between the number of traps with at least one egg and the total units installed and successfully retrieved. The EDI is the ratio between the number of eggs totalized for a given area by the number of ovitraps respective to the area^6^. In the present study, we use EDI calculated by neighborhoods since this index has higher discretization than OPI, which could be helpful for dengue incidence forecasting purposes.

### Heat map visualizations and scatter plots of socioeconomic variables

Heat maps based on EDI and dengue occurrence were used for visualizing the variation and dynamics of vector incidence and dengue cases (Fig. 1) throughout the monitored period. For the heat maps, rows indicate neighborhoods, columns indicate weeks, and the colors indicate relative values for the respective variables visualized. In addition, ovitrap indexes and dengue occurrence visualized as colored heat maps allowed us to obtain an easily interpretable image for gaining insights on possible associations for fluctuations in the number of Aedes eggs and dengue outbreaks. For the PCA presented in the right upper panel of Fig. 1, the variables population, total income by neighborhood, and income per capita were used. All of them were obtained from the last census performed in Brazil (2010).

### Models for dengue incidence forecasting

Several conventional models have been used in the literature for dengue time series forecasting, for instance, Artificial Neural Networks, Random Forest, Lasso Regression, Generalized Additive Models, and Autoregressive Models^19,35–37^. However, recent advances in Deep Learning (DL) methods have shown the remarkable performance of these algorithms in different fields of applications^38^, being Convolutional Neural Networks (CNNs) particularly popular for image analysis and computer vision^39^, and Long-Short Term Memory (LSTM) for sequence and time series analysis^40^. Specifically, LSTM is an excellent candidate model for dengue forecasting, and it has been used for this task to outperform conventional machine learning methods^19^. This section briefly explains LSTM fundamentals and describes how the models were configured for dengue forecasting.

Long Short-Term Memory Networks (LSTMs) are a particular type of Recurrent Neural Networks (RNN), which can learn long-term dependencies while dealing with the vanishing/exploding gradient problem^40,41^. LSTM are used in several applications related to sequential data, such as Natural Language Processing, time series prediction, computer vision, among others^40^. The architecture for all the LSTM networks trained in the study was the same, and it was composed of (1) an input layer, (2) an LSTM layer with 100 hidden units, (3) a fully connected layer, and (4) a regression layer. The networks were trained for 250 epochs, using a mean squared error (MSE) loss function and a Nesterov Adam optimizer, similar to the LSTM model trained in^19^.

### LSTM for forecasting dengue aggregate values for Natal

Aggregate values for dengue incidence and ovitrap data were used for training LSTM models. The target for forecasting will be either dengue incidence or EDI for the subsequent week. The model input will be represented as D when the predictor is dengue incidence or O when the input to the model is ovitrap index, that is, EDI. Then, dengue incidence (D) or ovitrap index – EDI (represented by O) were used as the target for prediction. The models were trained with the last sample for the predictor or the last three samples for the predictor. The nomenclature D → D means that the model was trained with dengue incidence as a predictor (past samples) and dengue incidence (subsequent week) as a target. Other possible combinations are D → O, O → D, and O → O. Models trained for ovitrap data forecasting were also evaluated for complementing the discussion described in the following sections.

### Computing accumulated values for 1-year sliding windows

Based on the times series of Precipitation, EDI, Dengue Incidence, and Dengue Hospitalizations for monthly samples (Fig. 5), the Accumulated values were computed for a 1-year sliding window, depicted in Fig. 6. The method used to compute accumulated values for one-year sliding windows is detailed in Supplementary Fig. 4. Fundamentally, for a given time series $x$, all samples for a 1-year length window are summed up, obtaining accumulated values corresponding to that 1-year window. Hence, by sliding the 1-year window and then computing the respective accumulated values, a new time series $y$ was obtained, as presented in the following equation:

$$ y\left( j \right) = \sum\limits_{{i = j}}^{{j + 11}} {x\left( i \right),j = {\text{0}},{\text{1}}, \ldots 36}  $$

To estimate the relationship between the time series analyzed, either for a series of monthly samples or for accumulated 1-year window series, the Spearman correlation coefficient^42^ was calculated between all pairwise time series combinations. The relationship between the mentioned time series was estimated by calculating the correlation coefficient between 25 samples selected randomly (100 times) for all possible pairwise combinations of the four-time series.

## Data and code availability

The database analyzed in this study as well as the code for the prediction models are publicly available online. The database is available at: https://zenodo.org/record/6408362 .

The source code of the prediction models is available in the repository: https://github.com/danielemontenegro/dengueknowledgePaper.git

## Supplementary Information

Supplementary Information.
