# Supplementary material for: Data-driven computational intelligence applied to dengue outbreak forecasting: a case study at the scale of the city of Natal, RN-Brazil
Source: Sci Rep. 2022 Apr 21;12:6550. doi: 10.1038/s41598-022-10512-5 (PMC9023501; doi:10.1038/s41598-022-10512-5)
Supplement: Supplementary file 1 — Supplementary Information. [file 41598_2022_10512_MOESM1_ESM.pdf]

Scientific Reports

Data-Driven Computational Intelligence Applied to Dengue Outbreak Forecasting: a case study at the scale of the city of Natal, RN-Brazil.

Ignacio Sanchez-Gendriz[1,3*], Gustavo Fontoura de Souza[2], Ion G.M. de Andrade[1], Adrião Duarte Doria Neto[3], Alessandre de Medeiros Tavares[4], Daniele M. S. Barros[1], Antonio Higor Freire de Morais[2], Leonardo J. Galvão-Lima[1], Ricardo Alexsandro de Medeiros Valentim[1]

[1]Laboratory for Technological Innovation in Health (LAIS), Hospital Universitário Onofre Lopes, Federal University of Rio Grande do Norte (UFRN), Natal/RN, Brazil; [2]Advanced Nucleus of Technological Innovation (NAVI), Federal Institute of Rio Grande do Norte (IFRN), Natal/RN, Brazil, [3]Department of Computer and Automation, UFRN, Natal/RN, Brazil; [4]Municipal Health Department, Zoonoses Control Center, Natal/RN, Brazil.

[*] *Correspondence Author: ignaciogendriz@gmail.com*

Abstract

Dengue is recognized as a health problem that causes significant socioeconomic impacts throughout the world, affecting millions of people each year. A commonly used method for monitoring the dengue vector is to count the eggs that Aedes aegypti mosquitoes have laid in spatially distributed ovitraps. Given this approach, the present study uses a database collected from 397 ovitraps allocated across the city of Natal, RN – Brazil. The Egg Density Index for each neighborhood was computed weekly, over four complete years (from 2016 to 2019), and simultaneously analyzed with the dengue case incidence. Our results illustrate that the incidence of dengue is related to the socioeconomic level of the neighborhoods in the city of Natal. A deep learning algorithm was used to predict future dengue case incidence, either based on the previous weeks of dengue incidence or the number of eggs present in the ovitraps. The analysis reveals that ovitrap data allows earlier prediction (four to six weeks) compared to dengue incidence itself (one week). Therefore, the results validate that the quantification of Aedes aegypti eggs can be valuable for the early planning of public health interventions.

Keywords: Ovitraps, Dengue, Deep Learning, LSTM

Supplementary figures.

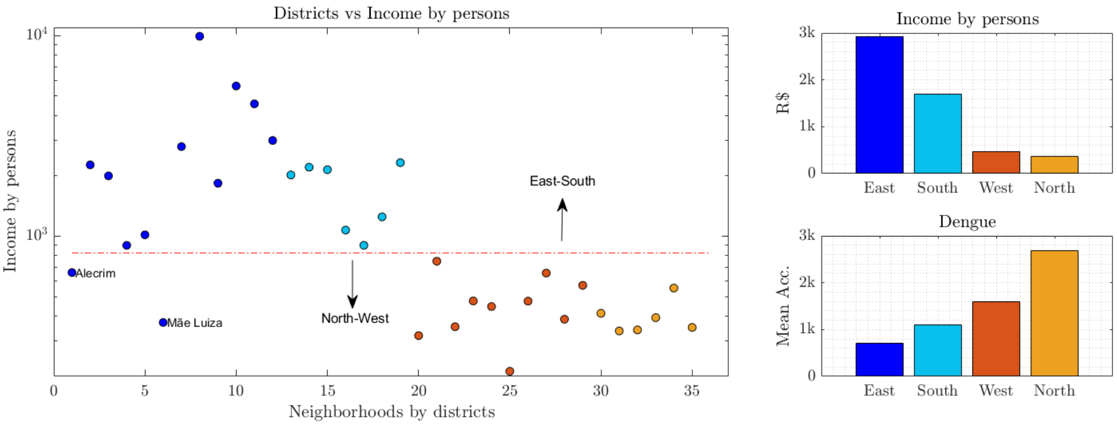

Supplementary Figure 1. Differentiating districts of Natal's city by income and mean accumulated dengue incidence. A) A scatterplot illustrating that Natal neighborhoods can be almost perfectly separated into two groups: Group 1 neighbourhoods, East and South districts and Group 2 neighbourhoods, North and West districts. The exceptions are the low-income neighborhoods 'Mãe Luiza' and 'Alecrim' that regionally belong to the East-South group, but have socioeconomic profiles compatible with the North-West group. B) Bar graphs for Income per capita and Dengue Incidence for the districts of Natal. It is worth noting that districts with the lowest income have the highest Dengue Incidence.

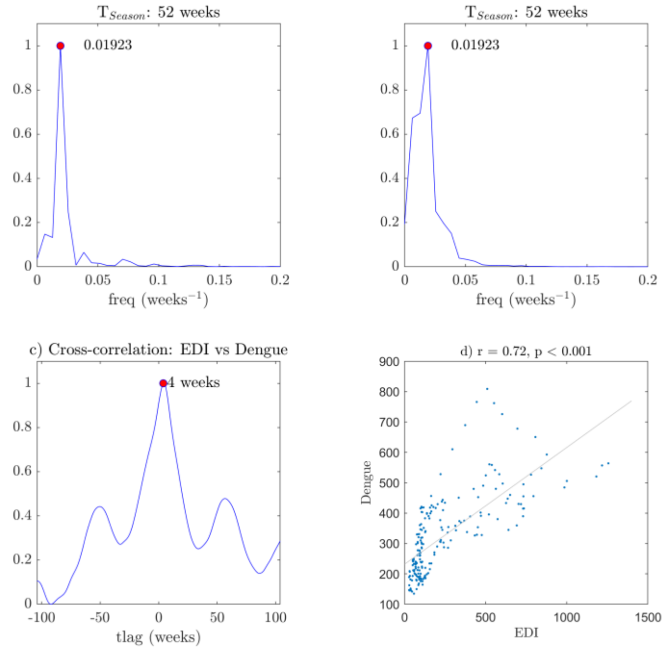

Supplementary Figure 2. Estimating periodicities for Dengue incidence and EDI time series and time lag between them; a) DFT for mean EDI time series, the estimated periodicity was 52 weeks. b) DFT for mean Dengue Incidence time series, the estimated periodicity was 52 weeks. c) time lag estimated by cross-correlation between mean EDI and mean Dengue Incidence was 4 weeks. d) Pearson correlation coefficient (r) and its p-value computed for the linear relation between mean EDI and Mean Dengue Incidence. Note: the period for seasonality (T_{season}) was estimated as $1/f_{peak}$, see panels a) and b).

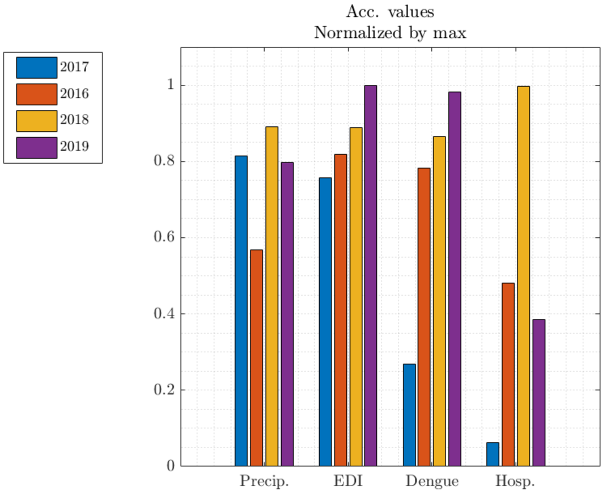

Supplementary Figure 3. Bar graph of the normalized accumulated values by years from 2016 to 2019 at Natal's city. The variable illustrated are Precipitation (Precip.), Egg Density Index (EDI), Dengue Incidence (Dengue) and Dengue Hospitalizations (Hosp.).

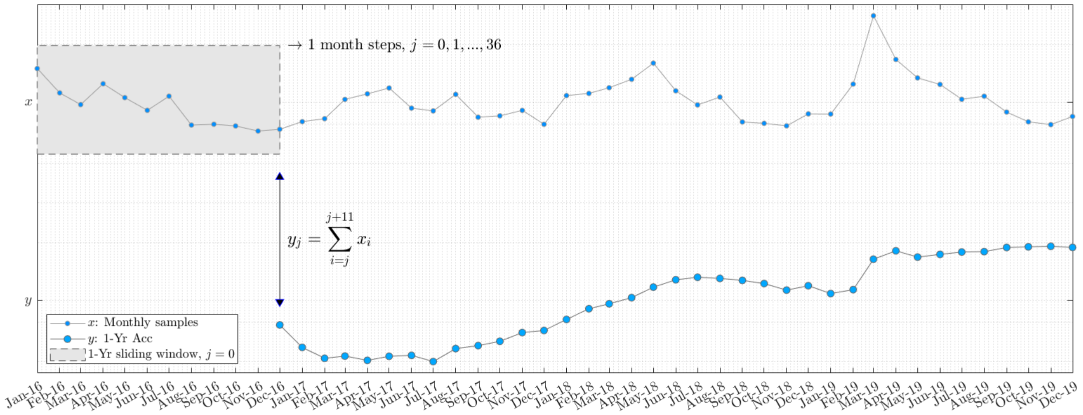

Supplementary Figure 4. Representation of the procedure used to calculate accumulated values for 1-year sliding windows. The time series represented by x is used to calculates accumulated values obtained from the sum of the samples within 1-year windows. These windows slides with a one-month step, from December 2016 to December 2019. The figure represents the first sliding window, which includes the samples of x between January 2016 and December 2016. A double arrow indicates both the last sample of the 1-year window, as well as the accumulated value for that window.

$$y(j) = \sum_{i=j}^{j+11} x(i)\,, j = 0, 1, \ldots 36$$

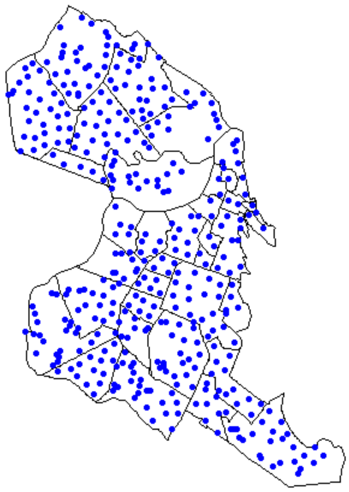

Supplementary Figure 5. Ovitrap distribution at Natal city. The map was generated using the free software R, version R x64 4.1.2 (https://www.r-project.org/) using free shapefile files available on the IBGE maps portal website (https://portaldemapas.ibge. gov.br).
